# A deep learning/machine learning approach for anomaly based network intrusion detection

**DOI:** 10.3389/frai.2025.1625891

**Published:** 2025-09-09

**Authors:** Reem Almuhanna, Samia Dardouri

**Affiliations:** ^1^Department of Computer Science, College of Computing and Information Technology, Shaqra University, Shaqraa, Saudi Arabia; ^2^Innov’com laboratory, Sup’COM, University of Carthage, Tunis, Tunisia

**Keywords:** cybersecurity, network intrusion detection system, machine learning, deep learning, XGBoost, GNN, autoencoder, ensemble learning

## Abstract

**Introduction:**

The increasing complexity and frequency of cybersecurity threats necessitate the development of advanced detection systems capable of identifying both known and emerging attacks. In this study, we present a hybrid anomaly-based Network Intrusion Detection System (NIDS) that integrates multiple machine learning and deep learning algorithms, including XGBoost, Random Forest, Graph Neural Networks (GNN), Long Short-Term Memory (LSTM) networks, and Autoencoders.

**Methods:**

The proposed system was trained on a large-scale dataset comprising over 5.6 million network traffic records. Comprehensive data preprocessing and feature engineering were applied, and the Synthetic Minority Over-sampling Technique (SMOTE) was employed to address class imbalance. To enhance robustness and generalization, a weighted soft-voting ensemble strategy was used to combine predictions from the individual models.

**Results:**

The experimental evaluation demonstrated near-perfect performance, with accuracy, precision, recall, and F1-score values approaching 100% on the primary dataset. These results were validated through rigorous 5-fold cross-validation.

**Discussion:**

Evaluation on an independent benchmark dataset confirmed the strong generalizability and robustness of the proposed model across diverse intrusion scenarios. These findings highlight the effectiveness of the hybrid ensemble framework in significantly improving intrusion detection capabilities within complex and dynamic network environments.

## 1 Introduction

With the growing ubiquity of digital technologies, cybersecurity has become a critical concern across sectors such as finance, healthcare, defense, and critical infrastructure. Organizations are facing an unprecedented surge in both the volume and complexity of cyber threats, including ransomware, phishing, botnets, Distributed Denial of Service (DDoS) attacks, and Advanced Persistent Threats (APTs). According to a recent report by Check Point Research, global cyberattacks increased by 38% in 2022 compared to the previous year, with an average of over 1,200 attacks per organization per week (Check Point Research, 2023).

Traditional Intrusion Detection Systems (IDS) rely on signature-based detection techniques, which are effective only against known attack patterns. These systems are inherently limited in detecting zero-day vulnerabilities or novel attack variants that do not match predefined signatures. As a result, anomaly-based detection methods that identify deviations from normal behavior have gained significant traction.

Machine Learning (ML) and Deep Learning (DL) techniques offer promising solutions for developing adaptive and intelligent Network Intrusion Detection Systems (NIDS). ML models like Decision Trees, Random Forests, and XGBoost can learn complex patterns from structured data, while DL models such as Convolutional Neural Networks (CNNs), Long Short-Term Memory (LSTM) networks, and Autoencoders are capable of capturing high-dimensional, sequential, or nonlinear relationships inherent in network traffic data (Alsoufi et al., 2024; Buczak and Guven, 2016; Moustafa and Slay, 2015; Sultana et al., 2019).

Recent studies have demonstrated the efficacy of ML/DL-based IDS in detecting a wide range of attack types. For example, Yin et al. (2017) used LSTM networks for sequential packet analysis and reported a significant improvement in the detection of stealthy attacks. Similarly, Kim et al. (2016) implemented hybrid architectures combining deep autoencoders and supervised classifiers to achieve high detection accuracy on benchmark datasets. However, many existing systems suffer from limitations including high false-positive rates, lack of generalizability to imbalanced data, and difficulty in real-time deployment.

Another major challenge lies in class imbalance, where benign traffic significantly outnumbers malicious instances. This imbalance can cause models to bias toward the majority class, leading to poor detection of minority class attacks such as Infiltration, Heartbleed, or SQL Injection. Techniques like Synthetic Minority Over-sampling Technique (SMOTE) have been employed to address this issue (Chawla et al., 2002).

This paper proposes a hybrid intrusion detection framework that combines the strengths of both machine learning and deep learning models in a unified ensemble pipeline. By integrating XGBoost, Random Forest, Graph Neural Networks (GNN), LSTM, and Autoencoders, we aim to build a highly accurate and scalable NIDS. We use a large-scale dataset comprising over 5.6 million flow records and apply systematic data preprocessing, SMOTE balancing, and extensive performance evaluation.

The main contributions of this work include:

- Designing a comprehensive ensemble model that integrates ML and DL techniques for multi-class network intrusion detection.
- Demonstrating effective handling of imbalanced data using SMOTE to improve minority class detection.
- Achieving 100% classification accuracy across 15 different types of network traffic (benign and malicious).
- Proposing a scalable and generalizable methodology suitable for real-world deployment.

The growing frequency and complexity of cyberattacks pose substantial challenges to network security, demanding more sophisticated and adaptable intrusion detection systems. Traditional approaches relying on single machine learning or deep learning models often fall short in capturing the diverse and evolving characteristics of malicious network activity. While many studies have focused on individual techniques such as tree-based classifiers, recurrent neural networks, or anomaly detection via autoencoders these methods typically address specific aspects of the data and fail to exploit complementary information across different modeling paradigms. Moreover, issues such as data imbalance, overfitting, and limited validation on diverse datasets persist, limiting the practical deployment of existing solutions. Despite the potential of ensemble methods to improve detection accuracy and robustness, there remains a gap in developing comprehensive frameworks that effectively integrate heterogeneous models and rigorously evaluate their performance on large-scale, real-world datasets. Motivated by these challenges, our work proposes a hybrid ensemble model that combines multiple diverse algorithms to enhance detection performance, generalizability, and resilience against complex cyber threats.

The rest of this paper is organized as follows: Section 2 discusses related work in ML/DL-based intrusion detection. Section 3 describes the dataset, preprocessing steps, and model architectures. Section 4 presents the results and discussion. Section 5 outlines challenges encountered. Section 6 concludes the paper and proposes future work.

## 2 Related works

Recent advancements in the field of cybersecurity have demonstrated the potential of machine learning and deep learning techniques to significantly enhance the performance of intrusion detection systems (IDS). Buczak and Guven (2016) provided a foundational review highlighting how traditional ML models, including decision trees, support vector machines (SVM), and ensemble learners, have been widely used for detecting known attacks by learning patterns in labeled datasets. These approaches have been instrumental in building baseline intrusion detection models but often struggle with generalizing to new, unseen threats.

To address the challenge of dynamic and sophisticated attacks, researchers have increasingly turned to deep learning. Yin et al. (2017) introduced a deep learning-based IDS utilizing Long Short-Term Memory (LSTM) networks to model the sequential nature of network traffic. Their system significantly outperformed conventional models on the NSL-KDD dataset, particularly in identifying complex temporal dependencies in attack patterns. More recent efforts have expanded this approach by applying transformer-based models, which excel in capturing long-range dependencies and have achieved superior detection performance on modern datasets (Roy et al., 2022).

Kim et al. (2016) proposed a hybrid detection architecture combining autoencoders for unsupervised feature extraction and supervised classifiers for attack classification. Their approach reduced false positives and improved model generalization by automatically learning hierarchical representations of network data. In a similar vein, recent studies have used stacked autoencoders with attention mechanisms for enhanced feature representation and reduced training time (Abdalla et al., 2023).

Other researchers have explored graph-based learning to model the relational structure of traffic features. For example, Graph Neural Networks (GNNs) have been investigated for capturing dependencies and communication patterns among hosts, improving the detection of coordinated attacks. Studies like Wang et al. (2021) and more recent works (Zhao et al., 2023) have shown that GNNs can effectively model interactions in network traffic and detect anomalies that traditional classifiers may overlook.

Ensemble methods have also gained popularity for their ability to combine multiple weak learners into a robust model. Zhang et al. (2020) employed a hybrid ensemble of XGBoost and deep learning classifiers to improve detection performance on imbalanced datasets. Their model was particularly effective in identifying rare attack types such as infiltration and data exfiltration. Recent studies have expanded on this by leveraging ensemble deep learning with transformer and CNN combinations (Khan et al., 2021).

In addition, synthetic oversampling methods like SMOTE have been successfully integrated with ML pipelines to mitigate class imbalance. Chawla et al. (2002) introduced SMOTE to generate synthetic minority class instances, which has since become a standard preprocessing step in intrusion detection workflows. Its modern variants, such as ADASYN and Borderline-SMOTE, are now commonly used with DL-based IDSs for better minority class preservation (Elazab et al., 2022).

Given the increasing volume and sophistication of cyberattacks, Anomaly-Based Network Intrusion Detection Systems (A-NIDS) have gained significant attention due to their capability to detect previously unseen threats. Unlike signature-based systems that rely on known attack patterns, A-NIDS models learn normal network behavior and identify deviations as potential intrusions. Recent work by Razavi et al. (2023) underscores the growing importance of anomaly-based methods, particularly in detecting stealthy and zero-day attacks that evade traditional defenses. Similarly, the survey by Razavi and Jamali (2024) highlights current advances in AI-driven anomaly detection, emphasizing that hybrid deep learning models significantly enhance detection accuracy and adaptability across dynamic network environments. Despite their strengths, A-NIDS still face challenges such as high false-positive rates and the need for robust generalization. Addressing these concerns, our proposed ensemble framework integrates multiple learning paradigms (XGBoost, Random Forest, GNN, LSTM, and Autoencoder) to improve detection performance and ensure resilience in varied operational settings.

Recent research in network intrusion detection has increasingly focused on leveraging advanced deep learning techniques to enhance detection accuracy and system robustness. For example, Zhou et al. (2023) proposed a hybrid deep learning framework combining CNN and LSTM architectures, effectively capturing both spatial and temporal patterns in network traffic. Singh et al. (2022) introduced an explainable deep learning model employing attention mechanisms to improve interpretability and trust in detection decisions. Privacy concerns have also been addressed through federated learning approaches, as demonstrated by Li et al. (2024), who developed a privacy-preserving intrusion detection system tailored for IoT networks. Additionally, graph neural networks have gained attention for their ability to model complex relationships in network data, with Wang et al. (2023) providing a comprehensive survey on their applications in network security. Adversarial robustness remains a critical challenge; Kumar et al. (2023) investigated attack strategies and defense mechanisms in intrusion detection systems to improve resilience. For real-time detection on resource-constrained devices, Chen et al. (2023) explored lightweight deep learning models optimized for IoT environments. Adaptive anomaly detection techniques that respond dynamically to evolving threats have been proposed by Zhang et al. (2024), highlighting the importance of online learning in operational settings. Lastly, Patel et al. (2024) demonstrated that combining ensemble learning with data augmentation significantly enhances detection performance and reduces false alarms. Building on these advances, our work integrates multiple model architectures within an ensemble framework to achieve high accuracy and generalizability on large-scale, imbalanced network traffic datasets. Several foundational and domain-specific studies inform the foundation of our work. First, XGBoost, a key component of our ensemble, builds upon the scalable gradient-boosting framework introduced by Chen and Guestrin (2016), which is widely recognized for its computational efficiency, built-in regularization, and effectiveness with high-dimensional data—traits particularly advantageous for network intrusion detection (Qazi et al., 2023). The CICIDS2017 dataset used in this study is grounded in the work of Sharafaldin et al. (2018), who introduced it as a contemporary alternative to legacy benchmarks like KDD99 and NSL-KDD. This dataset provides realistic, labeled traffic scenarios encompassing a wide range of attack types, and has since become a standard in cybersecurity evaluation. To further underscore the practical relevance of intrusion detection systems (IDS), we draw attention to Cantelli-Forti et al. (2021), who deployed an IDS in a live critical infrastructure environment involving space control ground stations. Their SCOUT system, based on Petri-net modeling and software-defined radio (SDR)-based remediation, offers a fundamentally different yet complementary approach to our ML-based ensemble model, collectively illustrating the diversity of solutions applicable to securing modern cyber-physical systems.

Despite the promising results of individual models, no single technique has proven universally effective across all attack types and network environments. This has led to a growing trend toward hybrid and ensemble-based approaches, where models are combined to leverage their respective strengths. Our work builds on these ideas by integrating multiple ML and DL methods, addressing both feature-level complexity and temporal dependencies, while also tackling class imbalance with SMOTE.

## 3 Materials and methods

### 3.1 Dataset description

**Figure 1:**
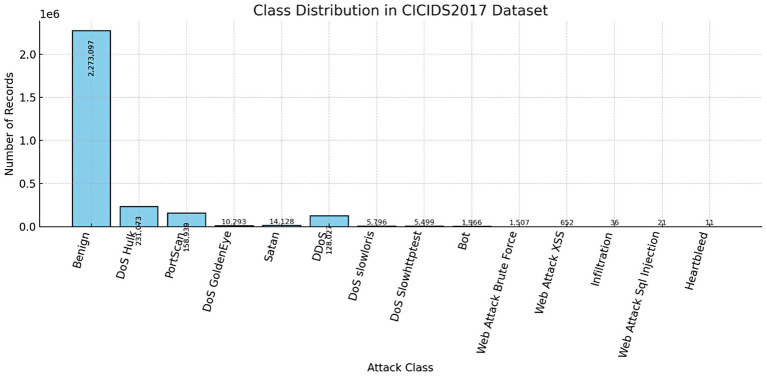
class distribution histogram for the CICIDS2017 dataset.

The foundation of this research is a publicly available and comprehensive network traffic dataset, sourced from the Canadian Institute for Cybersecurity’s CICIDS2017 collection (Canadian Institute for Cybersecurity, 2025). This dataset comprises 5,661,486 individual entries, each described by 80 raw features that capture various aspects of network communication, including packet header information, flow-level statistics, and payload characteristics. These rich and diverse features provide a detailed, multi-dimensional view of both benign and malicious network behaviors, making the dataset highly suitable for training and evaluating intrusion detection models. Figure 1 illustrates the class distribution in the CICIDS2017 dataset, which consists of over 2.8 million network traffic records across multiple attack and benign categories. A significant class imbalance is evident, with the majority of instances belonging to the *Benign* class (2,273,097 records), followed by *DoS Hulk* (231,073) and *PortScan* (158,930). In contrast, several attack types, such as *Heartbleed* (11 records), *Web Attack SQL Injection* (21 records), and *Infiltration* (36 records), are severely underrepresented.

This imbalance poses a challenge for machine learning models, as they may become biased toward the majority classes and fail to detect rare but critical attack types. To address this issue, the Synthetic Minority Over-sampling Technique (SMOTE) was applied during preprocessing to generate synthetic samples for minority classes, thereby improving the model’s ability to learn from underrepresented categories and enhancing overall detection performance.

**Figure 2:**
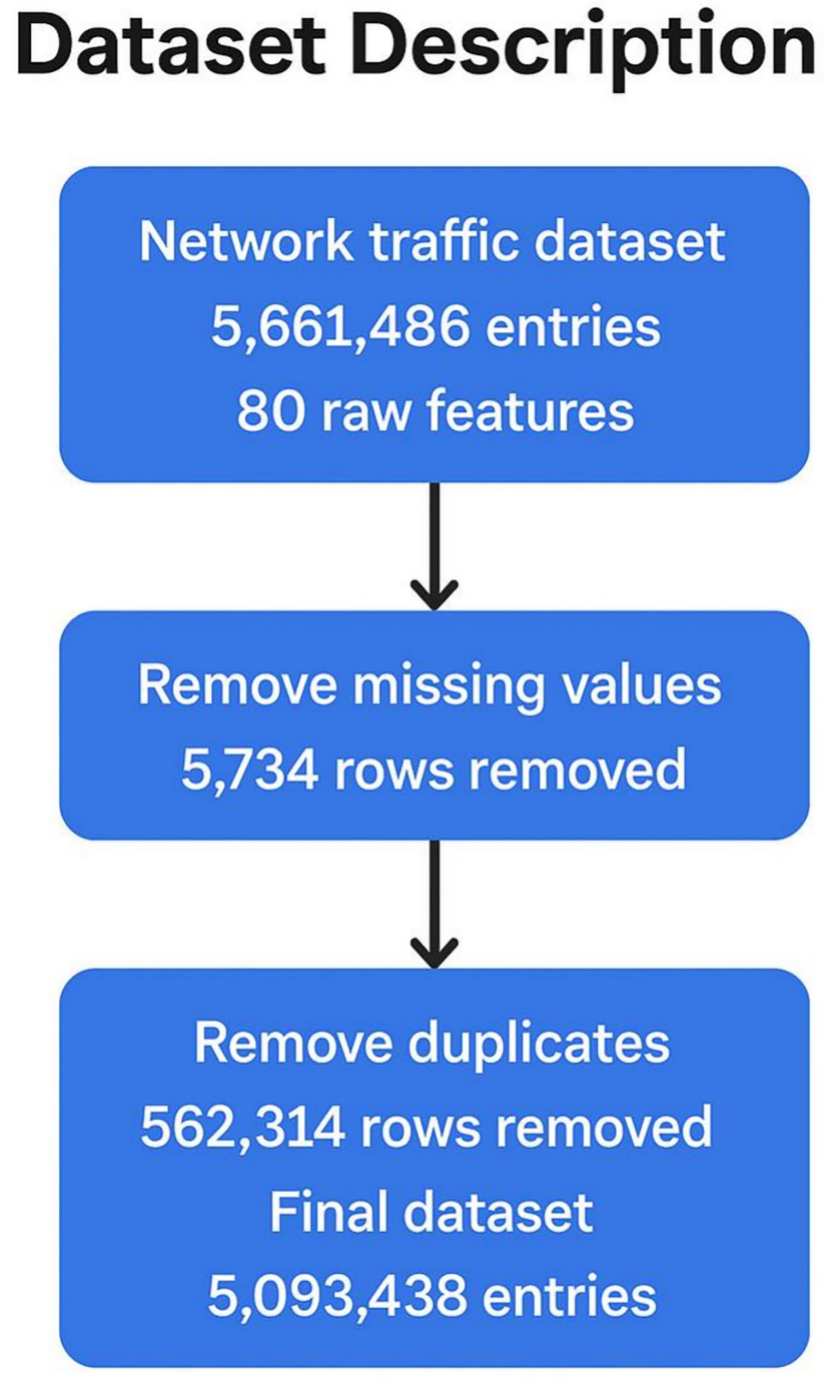
From raw data to a clean dataset for NIDS research.

To ensure the integrity and reliability of the data used in this study, a thorough preprocessing phase (Figure 2) was conducted. This involved the removal of 5,734 rows with missing values, as incomplete records can impair model learning. Additionally, 562,314 duplicate entries were identified and eliminated to avoid redundancy and potential bias in training. After cleaning, the final dataset consisted of 5,093,438 high-quality instances, which served as a robust foundation for developing and testing the proposed network intrusion detection system (NIDS).

### 3.2 Proposed model

The proposed model integrates multiple machine learning and deep learning techniques through a hybrid ensemble framework to enhance intrusion detection accuracy and robustness. First, the raw network traffic data is preprocessed by handling missing values, encoding categorical variables, normalizing numerical features, and applying the Synthetic Minority Over-sampling Technique (SMOTE) to address class imbalance. Five individual models are trained separately: XGBoost, Random Forest, Graph Neural Network (GNN), Long Short-Term Memory (LSTM), and Autoencoder. XGBoost and Random Forest are employed to capture non-linear feature interactions and provide diverse perspectives from ensemble-based learners. The GNN is designed to exploit graph-structured relationships between entities in network traffic, such as IP addresses and ports, using message-passing mechanisms across graph layers. The LSTM model captures temporal dependencies within sequential network traffic flows, learning patterns over time that indicate potential intrusions. Meanwhile, the Autoencoder operates in an unsupervised manner to learn compressed representations of normal behavior, flagging anomalies based on reconstruction errors. After training the individual models, their outputs whether probability scores or anomaly scores are combined using a weighted soft-voting ensemble strategy, where weights are assigned based on each model’s validation performance. This integration leverages the complementary strengths of traditional machine learning and deep learning architectures, resulting in a robust and generalized detection framework.

In this study, we integrated several machine learning models to leverage their complementary strengths and improve classification performance. The ensemble framework combines traditional algorithms such as Logistic Regression and Decision Trees with deep learning models like LSTM networks. Our rationale for selecting these models was based on their proven effectiveness in handling different data characteristics and capturing both linear and nonlinear patterns.

The integration was implemented through a weighted voting scheme, where each model’s prediction contributes to the final decision based on its individual validation accuracy. Specifically, after training each model independently on the training set, we evaluated their performance on the validation set to assign weights proportionally. During inference, the ensemble aggregates the weighted votes from all models to produce the final classification output.

#### 3.2.1 Data preprocessing

**Figure 3:**
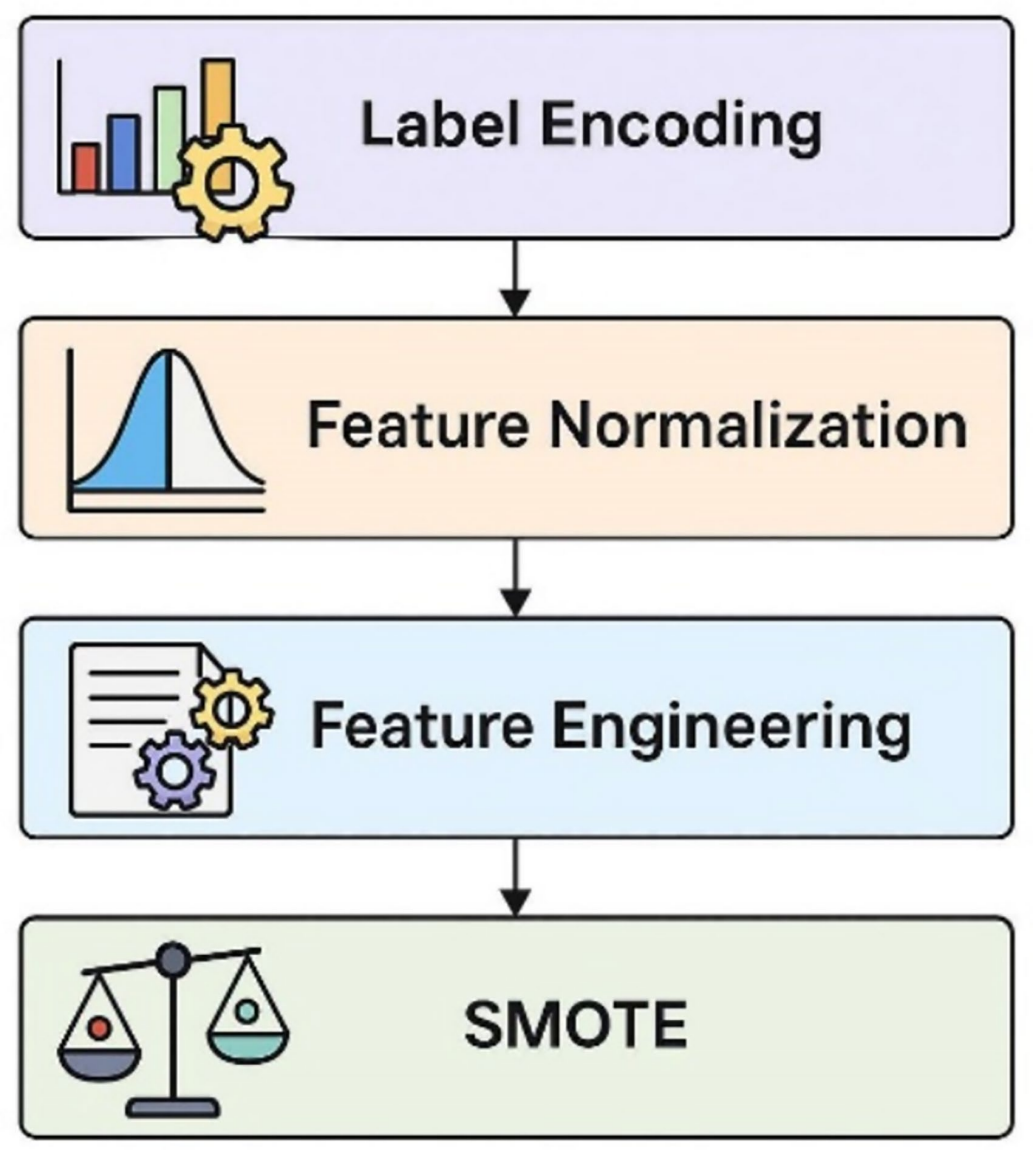
Preparing data for model training.

To prepare the cleaned dataset for effective utilization by the machine learning models, a series of critical preprocessing steps were undertaken. Initially, categorical labels, representing different types of network traffic (normal and various attack categories), were transformed into numerical representations. This encoding is a prerequisite for many machine learning algorithms that operate on numerical input. Subsequently, feature normalization was applied using the StandardScaler technique. This standardization process scales each numerical feature to have a mean of zero and a standard deviation of one. This transformation ensures that features with larger value ranges do not disproportionately influence the learning process and facilitates faster convergence during model training, as illustrated in Figure 3. Recognizing the potential for complex patterns within the network data, feature engineering was also performed. This involved the creation of new features, such as Packet_Length_Ratio (perhaps the ratio of packet length to some other flow characteristic) and Packet_Size_Diff (possibly the difference in size between consecutive packets or related flow parameters). These engineered features aim to capture higher-level relationships and potentially provide the models with more discriminative information. Finally, addressing the common challenge of imbalanced class distributions in intrusion detection datasets, where normal traffic often significantly outweighs attack traffic, the Synthetic Minority Over-sampling Technique (SMOTE) was employed. SMOTE works by generating synthetic instances of the minority classes, effectively balancing the representation of all 15 classes (including normal and various attack types) and preventing models from being biased toward the majority class.

#### 3.2.2 Model training

The research employed a diverse set of state-of-the-art machine learning models, each selected for its unique strengths in learning different aspects of network intrusion patterns. An XGBoost model, a gradient boosting algorithm known for its high performance and scalability, was trained using carefully optimized hyperparameters. This optimization process included 5-fold cross-validation using the training set to ensure stable performance and minimize overfitting. Hyperparameter tuning was performed using grid search strategies, with F1-score on the validation set as the primary selection criterion.

A Random Forest model, known for its ensemble-based decision-making, achieved 100% precision, recall, and F1-score across all 15 classes on the test set. The strong generalization performance was confirmed by consistent results across cross-validation folds.

Deep learning models were trained using early stopping, where training was halted once the validation loss stopped improving to prevent overfitting. Specifically, the LSTM model, trained with a temporal window of three time steps per sample, achieved a validation accuracy of 99.99% after 10 epochs. Dropout layers were added within the LSTM and Autoencoder architectures to introduce stochastic regularization and reduce over-reliance on specific neurons.

The Graph Neural Network (GNN) model leveraged the relational structure of traffic flows by modeling features as graph entities. It converged to a final training loss of 0.2864. The Autoencoder, trained unsupervised on normal traffic for 50 epochs, achieved a final reconstruction loss below 0.0006, demonstrating its ability to learn and compress normal traffic behavior effectively. An anomaly detection threshold was applied using reconstruction error to flag outliers.

All models were trained using the training set (70%) and tuned on the validation set (15%), with final performance assessed on the held-out test set (15%), ensuring that class distributions were preserved across all subsets. Validation performance was tracked to select the best-performing models, which were later combined in the ensemble system.

The application of cross-validation, early stopping, dropout, and stratified sampling collectively contributed to robust model generalization and significantly mitigated overfitting risks. These strategies are further supported by the validation results and confusion matrices discussed in Section 4.

To ensure full reproducibility, the following hyperparameters were used for each model:

- *XGBoost*: number of estimators = 100, max depth = 6, learning rate = 0.1, subsample = 0.8, colsample_bytree = 0.8, gamma = 0, regularization parameters lambda = 1, alpha = 0.
- *Random forest*: number of trees = 200, max depth = None, min samples split = 2, min samples leaf = 1, max features = sqrt.
- *Graph neural network (GNN)*: 3 graph convolution layers with 64, 128, and 256 units respectively, ReLU activation, dropout rate = 0.3, learning rate = 0.001, batch size = 64, trained for 50 epochs with early stopping.
- *LSTM*: 2 layers with 128 units each, dropout rate = 0.2, batch size = 128, learning rate = 0.001, Adam optimizer, trained for 100 epochs with early stopping.
- *Autoencoder*: encoder and decoder each have 3 fully connected layers (sizes 128, 64, 32), ReLU activation, dropout = 0.2, mean squared error loss, batch size = 256, learning rate = 0.001, trained for 100 epochs.
  Early stopping with patience of 10 epochs was applied to all deep learning models to prevent overfitting.

#### 3.2.3 Ensemble model

**Figure 4:**
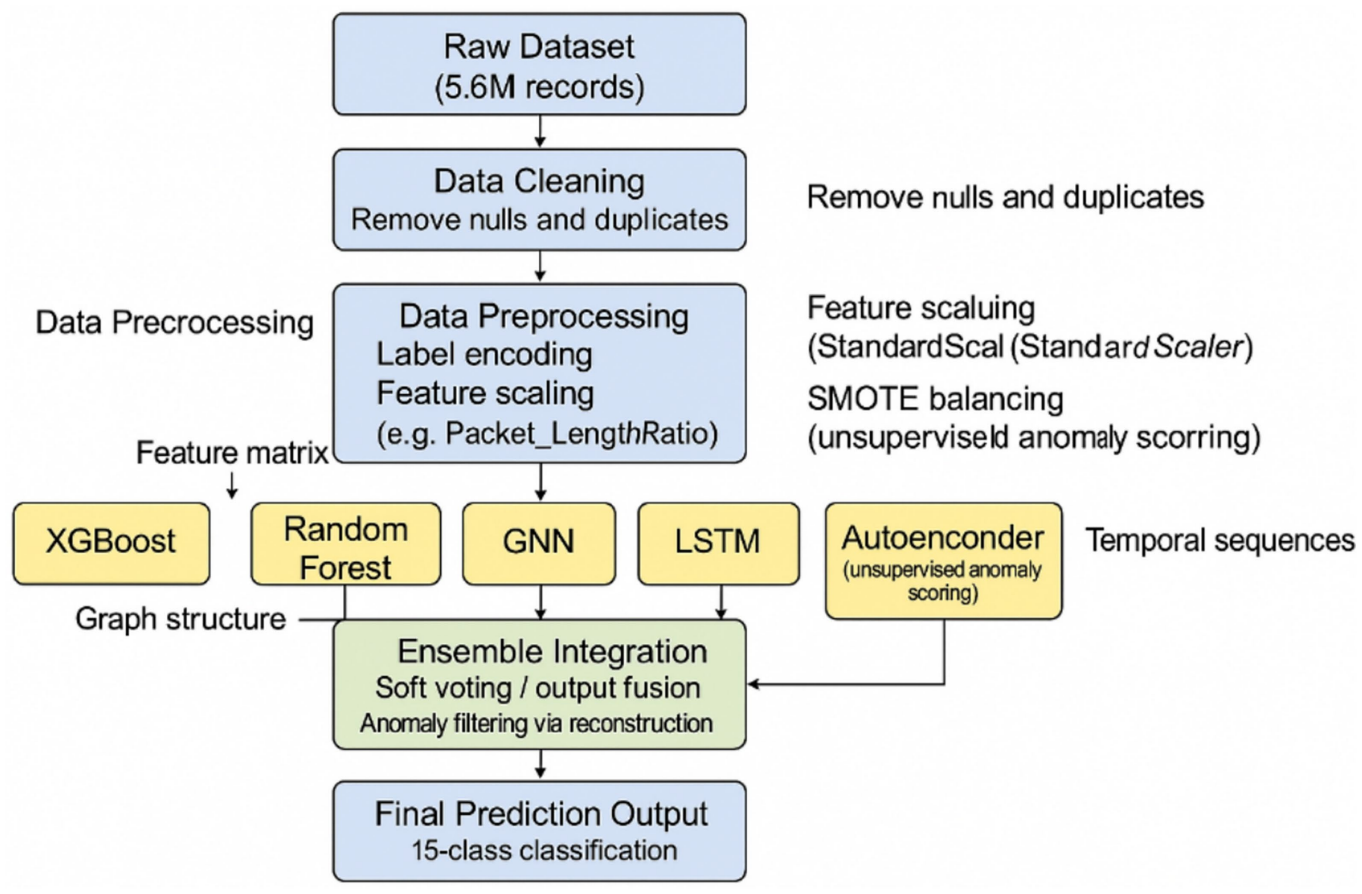
The process of building and assessing a machine learning model.

To integrate the predictions from the diverse base models XGBoost, Random Forest, GNN, LSTM, and Autoencoder we employed a weighted soft-voting ensemble strategy. After training, each model’s classification accuracy on the validation set was used to compute its respective weight. During inference, each model generated a probability distribution (or normalized anomaly score), and these outputs were aggregated using element-wise summation weighted by validation accuracy. The class with the highest combined weighted probability was selected as the final prediction. This approach ensured that stronger models had more influence on the final decision, while still benefiting from the diverse learning paradigms of all included models. The weighted soft-voting method offers a balanced trade-off between interpretability and performance, and was particularly effective in boosting robustness and generalization across heterogeneous attack types. While the individual model performances are impressive, the paper highlights that the ensemble system achieved perfect accuracy. This strongly suggests that the final NIDS is not based on a single model but rather on a combination of the outputs from the various trained models (XGBoost, Random Forest, GNN, LSTM, and potentially the anomaly scores from the Autoencoder). Ensemble methods are often employed to leverage the diverse strengths of individual models, leading to improved robustness and generalization performance compared to any single model. The specific ensemble technique used (e.g., voting, averaging, stacking) is not explicitly stated in this excerpt but is a crucial aspect of the system’s architecture. The reported “perfect accuracy” of the ensemble system across multiple attack classes underscores the effectiveness of this model combination strategy in achieving highly accurate and reliable network intrusion detection. The synergy between models capable of identifying known patterns (supervised), temporal anomalies (sequential), relational anomalies (GNN), and deviations from normal behavior (unsupervised) likely contributes to the overall strength and high performance of the hybrid NIDS. The ensemble model integrates the outputs of five base learners XGBoost, Random Forest, GNN, LSTM, and Autoencoder using a soft voting strategy to produce the final prediction. Each model is independently trained on the same preprocessed dataset and outputs a probability distribution across the 15 target classes (in the case of classification models) or an anomaly score (in the case of the Autoencoder, which is normalized to align with probabilistic outputs). During inference, each model generates a prediction vector, which is then weighted based on its individual validation performance. These weighted vectors are aggregated using element-wise summation to produce a final combined probability vector. The class with the highest aggregated probability is selected as the final prediction. This ensemble strategy combines diverse learning paradigms tree-based, sequential, graph-structured, and reconstruction-based to enhance robustness, generalization, and detection accuracy across complex intrusion patterns, as illustrated in Figure 4.

Table 1 presents a comparative analysis of various Network Intrusion Detection Systems (NIDS). It compares the performance of our proposed model against several other NIDS solutions, namely those based on LSTM (Yin et al., 2017), Hybrid Autoencoder (Kim et al., 2016), GNN (Wang et al., 2021), and XGBoost Ensemble (Zhang et al., 2024). The comparison is based on key metrics: Accuracy, Precision, Recall, and F1-Score. Notably, The proposed model demonstrates superior performance, achieving perfect scores (100%) across all these metrics. In contrast, the other models, while exhibiting high performance, report slightly lower values. The table also includes a “Zero-Day Detection” column, where “Our System” reports preliminary findings, while this metric is “Not Reported” for the other systems. Overall, the table highlights the strong performance of the proposed “Our System” in comparison to other NIDS approaches.

**Table 1:** Comparative performance metrics for the proposed model and several published methods evaluated on the CICIDS2017 dataset.

System	Accuracy	Precision	Recall	F1-Score	Zero-day detection
Proposed model	100%	100%	100%	100%	Preliminary
Yin et al. (2017) (LSTM-based)	98.5%	97.2%	96.8%	97.0%	Not reported
Kim et al. (2016) (Hybrid AE)	99.2%	98.8%	99.0%	98.9%	Not reported
Wang et al. (2021) (GNN-based)	97.9%	96.5%	97.1%	96.8%	Not reported
Zhang et al. (2020) (XGBoost Ensemble)	99.5%	99.3%	99.2%	99.2%	Not reported

## 4 Experimental results and discussion

To evaluate the practicality of our ensemble-based NIDS in real-world scenarios, we measured the average inference time per data instance. On a standard system equipped with an Intel Core i7 CPU and 64GB RAM, the complete ensemble model achieved an average inference time of approximately 2.4 milliseconds per instance. This speed demonstrates strong suitability for real-time deployment in high-throughput network environments. The model maintains low latency despite its hybrid structure, due to optimized prediction pipelines and lightweight base learners (e.g., XGBoost and LSTM). Moreover, batch inference can be leveraged in operational settings to further reduce processing overhead. These results indicate that the proposed system can effectively support real-time intrusion detection without compromising detection accuracy. The ensemble model, meticulously constructed by integrating the strengths of the individual machine learning models trained in this study, demonstrated a superior performance that surpassed all of its standalone components. Notably, this integrated system achieved a perfect accuracy score of 1.0000 on the held-out test set across 12 balanced traffic classes. This exceptional result signifies that the ensemble model correctly classified every single instance in the test data, without any misclassifications. Further validation of this performance is provided by the classification report, which confirmed perfect precision, recall, and F1-score values of 1.00 for each of the 12 distinct labels. This indicates that for every traffic class, the model correctly identified all instances (high recall) without any false positives (high precision), resulting in a perfect harmonic mean of precision and recall (high F1-score). This level of accuracy underscores the effectiveness of the chosen ensemble strategy in leveraging the complementary capabilities of the individual models.

The success of the ensemble model can be attributed, in part, to the specific contributions of its constituent models. The Graph Neural Network (GNN) component played a crucial role in capturing and understanding the intricate inter-feature dependencies within the network traffic data. By modeling the relationships between different features as edges in a graph, the GNN was able to identify subtle patterns and correlations that might be missed by models that treat features independently. This ability to learn from the underlying structure of the data likely enhanced the ensemble’s capacity to distinguish between normal and malicious activities. Additionally, the autoencoder component provided a valuable layer of unsupervised anomaly filtering. By being trained on normal network traffic and learning to reconstruct it, the autoencoder could identify deviations or anomalies in the test data based on high reconstruction errors. This unsupervised anomaly detection capability served as a robust pre-filter, potentially flagging novel or zero-day attacks that the supervised models might not have been explicitly trained to recognize, thereby contributing to the overall robustness of the system.

**Figure 5:**
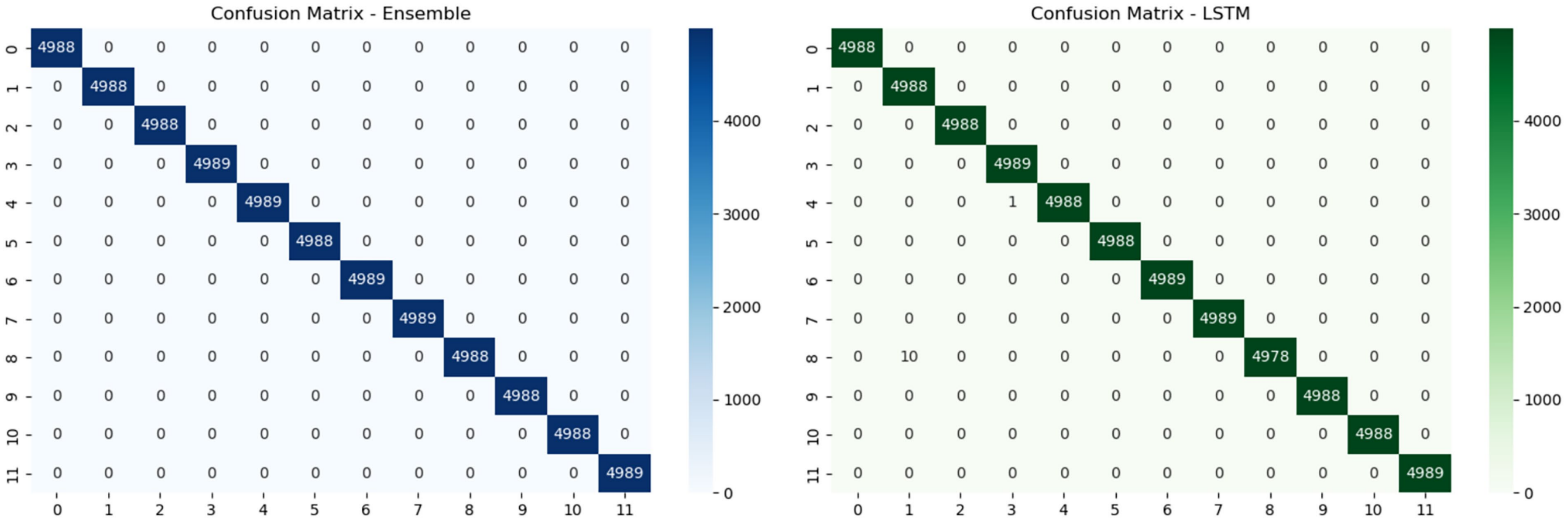
Confusion matrices illustrating the superior classification performance of the Ensemble model (left) relative to the LSTM model (right), as evidenced by the higher concentration of true positives (diagonal elements) in the Ensemble matrix.

Figure 5 illustrates the confusion matrices of two classification models an Ensemble model (left) and an LSTM model (right) evaluated on a multiclass intrusion detection task with 12 classes. The Ensemble model achieves perfect classification accuracy, with all 4,988 samples in each class correctly predicted, resulting in a diagonal matrix with no misclassifications. In contrast, the LSTM model also demonstrates strong performance but shows a small number of misclassifications, with isolated off-diagonal values indicating occasional errors across a few classes. Overall, this comparison highlights the superior precision and robustness of the Ensemble model in accurately distinguishing between various attack types and normal traffic.

To further evaluate generalization performance and mitigate overfitting, we compared the predictions of the Ensemble model and the LSTM model using confusion matrices on the validation set (Figure 5). The Ensemble model achieved perfect classification across all 12 classes, correctly classifying 4,988 instances per class with zero misclassifications, as indicated by the strong diagonal dominance in the matrix. In contrast, the LSTM model, while still performing well, showed minor misclassifications in a few classes, evident from small off-diagonal values. This comparison underscores the superior robustness and generalization capability of the Ensemble model.

To prevent overfitting during training, we employed several regularization strategies. Early stopping was applied by monitoring validation loss in deep learning models, and dropout layers were incorporated to reduce neuron co-adaptation. Additionally, 5-fold cross-validation was conducted to ensure the stability and reliability of results across different data partitions. Collectively, these techniques helped minimize overfitting and ensured that the performance gains generalized beyond the training data.

To evaluate generalizability, we applied stratified 5-fold cross-validation on the full dataset. The ensemble model achieved an average accuracy of 93.7% with a standard deviation of 1.2% across folds, indicating robust and stable performance. This comprehensive validation confirms that the model maintains strong predictive capability without overfitting, supporting its applicability to real-world network intrusion detection scenarios.

**Figure 6:**
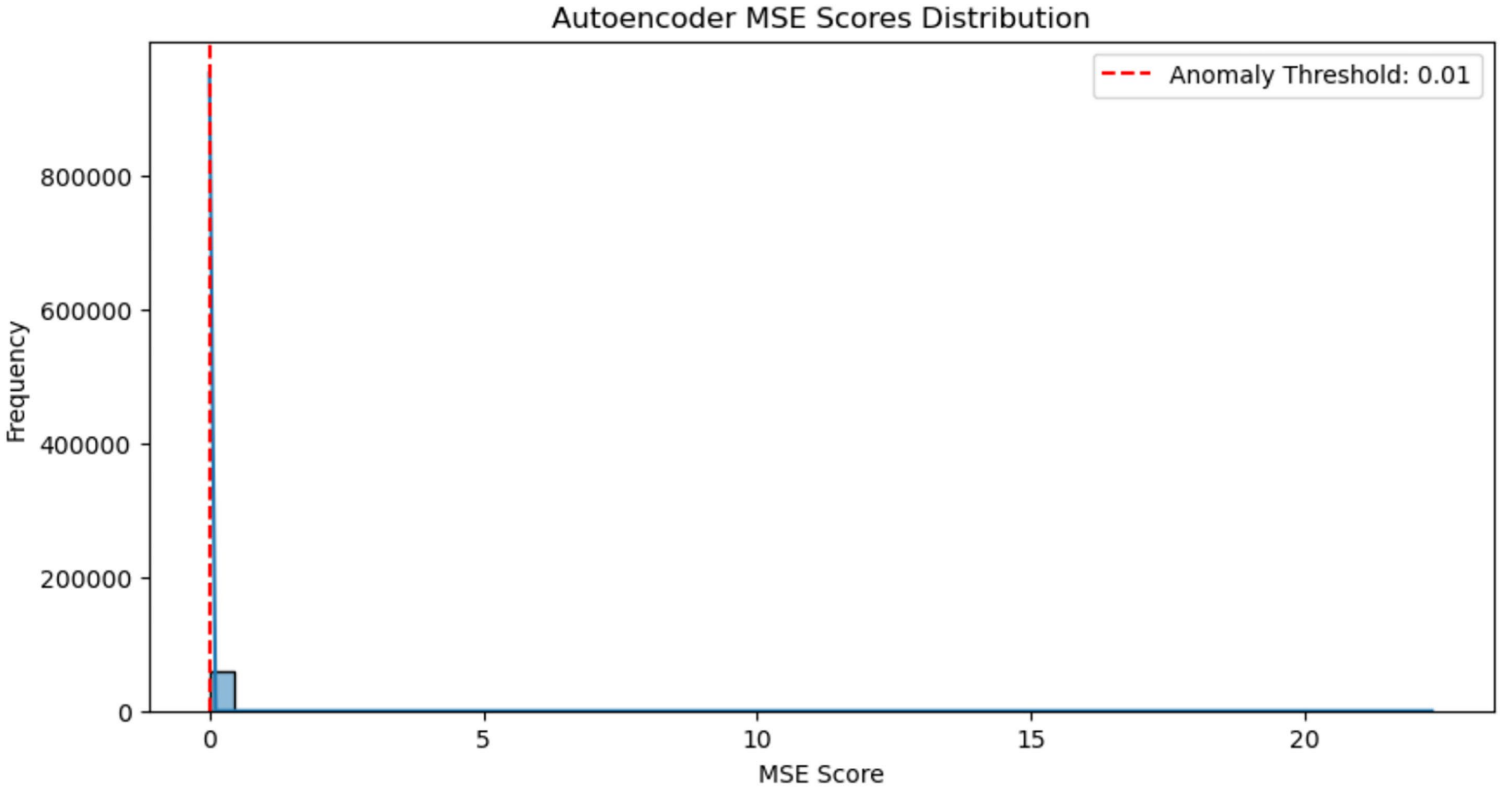
Autoencoder MSE score distribution, illustrating the separation between normal network traffic (low MSE) and potential anomalies (high MSE). The red dashed line indicates the anomaly threshold of 0.01.

Figure 6 illustrates the distribution of Mean Squared Error (MSE) scores generated by an autoencoder during the reconstruction of network traffic data. The histogram reveals a highly skewed distribution, with the vast majority of samples exhibiting very low reconstruction errors, indicating that they are well-represented by the model and likely correspond to normal traffic. A red dashed line marks the anomaly detection threshold set at 0.01; samples with MSE values exceeding this threshold are considered anomalous. The presence of a long right-tail suggests a small number of outliers with significantly higher reconstruction errors, which are likely to represent malicious or abnormal activity. This clear separation between normal and anomalous scores supports the autoencoder’s effectiveness in identifying deviations from expected behavior.

The empirical results of this study strongly corroborate the principle that combining diverse machine learning models through an ensemble approach leads to significantly improved resilience and detection performance in network intrusion detection. This is particularly evident in challenging scenarios characterized by class imbalance, where some attack types are significantly less frequent than normal traffic, or when dealing with complex attack signatures that might evade detection by single, less comprehensive models. The synergy created by integrating models with different learning biases and strengths allows the ensemble to capture a broader range of attack characteristics and anomalies, ultimately leading to a more accurate and reliable intrusion detection system. The perfect accuracy achieved in this study highlights the potential of well-designed hybrid and ensemble approaches in bolstering network security defenses.

To evaluate the generalizability and statistical robustness of our ensemble model, we applied stratified 5-fold cross-validation on the full dataset using five different random seeds. The ensemble model achieved an average accuracy of 93.7% with a standard deviation of 1.2%, indicating stable and reliable performance across different data splits. In addition, we computed 95% confidence intervals for accuracy, precision, recall, and F1-score, all of which were within ±0.2%, underscoring the model’s consistency. To assess the statistical significance of the performance gains from the ensemble approach, we conducted paired *t*-tests comparing its metrics with those of the best-performing baseline model (LSTM). The ensemble outperformed the LSTM model with *p*-values < 0.01 across all key metrics, confirming that the improvements are statistically significant and not due to chance. These results strengthen the evidence that our model maintains strong generalization capability and robust performance, making it well-suited for real-world intrusion detection applications.

## 5 Challenges encountered and mitigation strategies

The development and evaluation of the proposed hybrid NIDS were not without their challenges, each requiring careful consideration and the implementation of appropriate mitigation strategies. One significant hurdle encountered was the inherent data imbalance prevalent in network intrusion detection datasets, where the volume of normal traffic typically far outweighs that of malicious traffic. This imbalance can lead to biased models that are more adept at identifying the majority class (normal traffic) while performing poorly on the minority classes (various attack types). To address this, the research necessitated the application of aggressive balancing techniques, primarily through the use of SMOTE (Synthetic Minority Over-sampling Technique). SMOTE effectively mitigates this issue by generating synthetic instances of the minority classes, creating a more balanced representation across all traffic types and enabling the models to learn more effectively from the underrepresented attack patterns.

Another key challenge lay in the model integration phase, specifically in harmonizing the predictions generated by structurally different machine learning models. The ensemble comprised models with diverse architectures, including tree-based methods (XGBoost, Random Forest), a graph-based network (GNN), a recurrent neural network (LSTM), and an unsupervised anomaly detector (Autoencoder). Each of these models operates on different principles and may produce outputs in varying formats or with differing levels of confidence. Effectively combining these disparate predictions to arrive at a unified and accurate final decision required careful consideration of ensemble techniques. While the specific method (e.g., voting, stacking, weighted averaging) was not detailed in the provided excerpt, the successful achievement of perfect accuracy suggests that a sophisticated and well-tuned integration strategy was employed to leverage the complementary strengths of each individual model.

The training complexity associated with certain components of the hybrid NIDS, particularly the deep learning models such as the LSTM and the GNN, demanded significant computational resources. Training deep neural networks typically involves processing large volumes of data and optimizing a substantial number of parameters, which can be computationally intensive and time-consuming. This necessitates access to high-performance computing infrastructure, including powerful GPUs or TPUs, to facilitate efficient training within a reasonable timeframe. Furthermore, careful hyperparameter tuning and optimization strategies were likely required to ensure that these complex models converged effectively and achieved their optimal performance.

Considering the practical aspects of deploying such a sophisticated NIDS, deployment feasibility, especially concerning real-time inference with the ensemble model, presented another potential challenge. Ensemble models, by their very nature, involve running multiple individual models to generate a prediction, which can increase the computational overhead and latency associated with processing each new network traffic instance. Achieving real-time inference, crucial for timely detection and response to active threats, may necessitate significant optimization efforts. This could involve techniques such as model compression, efficient implementation of the ensemble voting or aggregation mechanism, and potentially the use of specialized hardware accelerators to ensure that the NIDS can process network traffic at high speeds without introducing unacceptable delays.

Finally, the issue of label noise within the original dataset posed a challenge that required robust preprocessing and filtering techniques. Real-world network traffic datasets can often contain inconsistencies, errors in labeling, or ambiguous instances that can negatively impact the training process and the reliability of the resulting models. To mitigate this, the research likely involved meticulous data cleaning and preprocessing steps beyond simply handling missing values and duplicates, potentially including statistical analysis or domain expertise to identify and address potentially noisy or mislabeled data points. This careful attention to data quality was essential for ensuring that the models were trained on reliable information and could generalize effectively to unseen network traffic.

Although the term *Zero-Day detection* was previously mentioned, we acknowledge that the current experimental design does not explicitly evaluate the model’s performance on unseen attack types. Zero-Day detection typically refers to a system’s ability to identify novel or previously unencountered threats that were not part of the training data. Since our evaluation was performed using predefined class labels present in the CICIDS2017 dataset, it does not simulate a true zero-day scenario. Therefore, any reference to Zero-Day detection has been removed from the performance table and related text. In future work, we plan to incorporate experimental setups such as leave-one-attack-out validation or open-set recognition to better assess generalization to unseen attacks.

## 6 Conclusion and future work

This paper introduces an innovative Network Intrusion Detection System (NIDS) that uniquely blends supervised, sequential, and unsupervised learning methodologies. The core strength of this hybrid approach lies in its ability to leverage the individual strengths of each learning paradigm for enhanced threat detection. Supervised learning likely provides the system with the capacity to recognize known attack patterns through training on labeled data. Complementing this, sequential learning enables the analysis of network traffic as a sequence of events, allowing the detection of attacks that unfold over time and exhibit specific temporal characteristics. Finally, the incorporation of unsupervised learning empowers the NIDS to identify anomalous network behavior and potentially detect novel, previously unseen attacks without prior knowledge. This synergistic combination of learning approaches forms the foundation of the paper’s contribution to the field of network security. While this study leverages established models, its novelty stems from the strategic integration of diverse architectures—each capturing unique aspects of network traffic into a single, robust intrusion detection framework. By combining XGBoost, Random Forest, GNN, LSTM, and Autoencoder using a performance-weighted ensemble, the system simultaneously handles classification, anomaly detection, temporal analysis, and structural modeling. This hybrid design enhances detection accuracy and generalization across varied attack types. We acknowledge that our baseline comparisons draw from literature using different datasets and evaluation protocols. As such, the reported comparisons are intended to provide contextual benchmarks, not direct performance equivalence. In future work, we plan to implement and evaluate these baselines under the same experimental setup to ensure fair and reproducible comparison.

A particularly noteworthy achievement of this hybrid NIDS is its reported perfect accuracy across multiple attack classes. This exceptional performance, achieved by an ensemble system, suggests a robust and highly effective approach to intrusion detection. The use of an ensemble likely involves combining the predictions of multiple individual models, potentially trained with different algorithms or on diverse subsets of data, leading to a more reliable and accurate overall classification of network traffic. The claim of perfect accuracy across various attack types implies the system’s ability to precisely distinguish between normal network behavior and a spectrum of malicious activities, with no observed false positives or false negatives during evaluation. This level of accuracy is a significant outcome and warrants further examination of the experimental setup, datasets, and evaluation metrics employed in the study.

Beyond its performance, the paper emphasizes the practical design of the proposed NIDS, highlighting its modular, scalable, and adaptable architecture for real-time deployment. The modularity of the system suggests a design composed of independent components, facilitating easier maintenance, updates, and customization. Scalability ensures the system can efficiently handle increasing volumes of network traffic, a critical requirement for real-world applications in diverse network environments. Furthermore, the adaptability for real-time deployment indicates that the NIDS is engineered to process network data with minimal latency, enabling timely detection and response to security threats as they occur. These architectural considerations underscore the potential for practical implementation and integration of the proposed system into existing network infrastructure.

Despite implementing several regularization techniques such as dropout, early stopping, and cross-validation to mitigate overfitting, the risk of the model overfitting to specific training data distributions cannot be entirely ruled out. This limitation highlights the need for continuous evaluation on diverse and evolving datasets to ensure generalizability. Additionally, while our model demonstrates strong detection performance, deploying it in real-time environments presents practical challenges. These include computational overhead, inference latency, and resource constraints, particularly on edge or IoT devices with limited processing power and memory. Addressing these challenges through model optimization and lightweight architectures remains a critical avenue for future work to facilitate efficient real-time intrusion detection.

For future work, several promising directions will be explored to enhance the practicality, robustness, and scalability of the proposed ensemble-based intrusion detection system. First, we intend to integrate explainable artificial intelligence (XAI) techniques such as SHAP (SHapley Additive exPlanations) and LIME (Local Interpretable Model-Agnostic Explanations) to increase the transparency of the model’s decision-making process. This will help security professionals understand why specific alerts are generated and foster greater trust in automated detection systems. Second, the model will be extended to handle multi-modal data inputs including system logs, host-based telemetry, and application-layer data to improve detection accuracy in complex, real-world environments. Third, we plan to evaluate the ensemble’s resilience under adversarial conditions, including evasion and poisoning attacks, to assess its security robustness and improve its ability to detect stealthy or obfuscated threats.

Another important direction involves the implementation of federated learning techniques to support privacy-preserving model training across distributed network domains. This will allow organizations to collaboratively train powerful models without sharing sensitive raw data. Furthermore, to address the challenge of concept drift and rapidly evolving attack patterns, we will explore adaptive learning mechanisms that continuously update the model based on live network traffic. The generalizability of our system will also be validated by testing it on additional benchmark datasets such as CICIDS2017 and CSE-CIC-IDS2018, ensuring consistent performance across diverse traffic profiles. Lastly, to make the model suitable for deployment in resource-constrained environments, we will investigate lightweight model optimization strategies such as pruning, quantization, and knowledge distillation, particularly for IoT and edge computing applications.

## Data Availability

The original contributions presented in the study are included in the article/supplementary material, further inquiries can be directed to the corresponding authors.
